# Risk factors associated with the progression and metastases of hindgut neuroendocrine tumors: a retrospective study

**DOI:** 10.1186/s12885-017-3769-4

**Published:** 2017-11-16

**Authors:** Yoichiro Okubo, Rika Kasajima, Masaki Suzuki, Yohei Miyagi, Osamu Motohashi, Manabu Shiozawa, Emi Yoshioka, Kota Washimi, Kae Kawachi, Yoichi Kameda, Tomoyuki Yokose

**Affiliations:** 1 Department of Pathology, Kanagawa Cancer Center, 2-3-2, Nakao, Asahi-Ku, Yokohama, Kanagawa 241-8515 Japan; 2 Molecular Pathology and Genetics Division, Kanagawa Cancer Center Research Institute, 2-3-2, Nakao, Asahi-Ku, Yokohama, Kanagawa 241-8515 Japan; 3 Department of Gastroenterology, Kanagawa Cancer Center, 2-3-2, Nakao, Asahi-Ku, Yokohama, Kanagawa 241-8515 Japan; 4 Department of Gastrointestinal Surgery, Kanagawa Cancer Center, 2-3-2, Nakao, Asahi-Ku, Yokohama, Kanagawa 241-8515 Japan

**Keywords:** Neuroendocrine tumor, Hindgut, Angiogenesis, Microvessel density, Lymphatic microvessel density, Lymphovascular invasion

## Abstract

**Background:**

The worldwide incidence of neuroendocrine tumors (NETs) has increased remarkably, with the hindgut being the second most common site for such tumors. However, the mechanisms underlying progression and metastasis of hindgut NETs are unclear. A retrospective study was conducted to elucidate these mechanisms.

**Methods:**

Clinicopathological data of cases of hindgut NET between April 1996 and September 2015 were analyzed, retrospectively. Patients with neuroendocrine carcinoma were excluded. Formalin-fixed paraffin-embedded tissues of hindgut NET cases were subjected to detailed morphometric and immunohistochemical analyses. Statistical analyses were performed using the non-parametric Mann-Whitney U test, Spearman’s correlation coefficient, and chi-squared test. Multivariate logistic regression analysis was conducted as appropriate for the data set.

**Results:**

Fifty-six hindgut NET cases were considered. Microvessel density and lymphatic microvessel density were identified as significant risk factors for venous and lymphatic invasion. There was a positive correlation between microvessel density and the maximum tumor diameter. Multivariate logistic regression analysis revealed that the maximum tumor diameter alone was an independent predictor of lymph node metastasis, whereas lymphovascular invasion and MVD was not the predictor of lymph node metastasis. There were no significant correlations between the Ki-67 labeling index and any of the parameters evaluated including age, sex, the maximum tumor diameter, venous invasion, lymphatic invasion, microvessel density, lymphatic microvessel density, and lymph node metastasis.

**Conclusions:**

Angiogenic mechanisms may play important roles in the progression of hindgut NET. Otherwise, the maximum tumor diameter alone was an independent predictor of lymph node metastasis in hindgut NETs. Moreover, our study raises the question of whether the presence of lymphovascular invasion, in endoscopically obtained hindgut NET tissues, is an absolute indication for additional surgery or not.

## Background

Neuroendocrine tumors (NETs) arise in many organs and the majority of them are gastroenteropancreatic neuroendocrine tumors (GEP-NETs) [1–3]. While the occurrence of GEP-NETs has been regarded relatively rare [4], a study recently reported a steady increase in the incidence and prevalence of GEP-NETs [1]. Globally, the midgut is the most common site of GEP-NETs; however, the fact that the hindgut is the second most common site could account for the remarkable increase in incidence [5, 6].

The World Health Organization (WHO) grading system for GEP-NETs was updated in 2010 [4]. This grading system is based on the proliferative activities of tumor cells (mitotic counts and Ki-67 labeling index). Indeed, both high levels of mitotic activity and Ki-67 immunoreactivity are associated with poor prognosis in perspective. Nevertheless, hindgut NET cases with relatively low levels of proliferative activities may have discordant tumor progression, invasion, metastasis, and/or overall prognosis [4, 7, 8]. To elucidate hidden risk factors for hindgut NETs, we previously conducted a pathological study using endoscopically resected specimens of hindgut NET and proposed that angiogenesis plays an important role in the initial phase (occurrence and progression) of this tumor [4]. To obtain a more detailed and accurate assessment of the mechanisms of hindgut NET progression and metastasis, we sampled a greater number of patients, including those who had undergone surgery.

## Methods

### Study design

In this retrospective study, data from patients with NET G1-G2 treated at our Institute between April 1996 and December 2015 was analyzed. We adopted a similar procedure as used previously, to identify cases of hindgut NETs [4]. Using the database system for the anatomic pathology ("EXpath" Laboratory Information Systems for Pathology, INTEC Inc, Tokyo, Japan.), we searched pathological records between April 1996 and December 2015, and subsequently retrieved the formalin-fixed paraffin-embedded (FFPE) tissue sections of the identified hindgut NET cases (including, tissue sections obtained from both endoscopic and surgical procedures). Data from patients with neuroendocrine carcinoma (NEC) were excluded because: (i) the clinical management of NEC is different [9], and (ii) studies have shown that colorectal NEC and hindgut adenocarcinoma have a similar mutation profile that differs from that of NET G1-G2 [10, 11].

### Clinicopathological data of identified hindgut NET cases

As previously reported [4], the clinicopathological data were analyzed for age, sex, tumor site, the maximum tumor diameter, depth of tumor invasion, lymphovascular invasion, the status of lymph node, and distant metastasis. The maximum tumor diameter was defined as largest tumor size based on macroscopic and pathological examination. Immunohistochemical examinations were also performed using antibodies against the following markers: CD31 (Leica, clone 1A10; 1:20 dilution), chromogranin A (Roche, clone LK2H10; 1:5 dilution), D2–40 (Roche, clone D2–40; 1:1 dilution), Ki-67 (Dako, clone MIB-1; 1:50 dilution), and synaptophysin (Roche, clone MRQ-40; 1:1 dilution). Tumor cells, which showed positive reactivity for synaptophysin and/or chromogranin A were analyzed in the present study (≥50% reactivity was defined as positive).

The Ki-67 labeling index was calculated using the Patholoscope image analysis software (MITANI Corporation, Japan, URL: http://www.mitani-visual.jp/en/products/bio_imaging_analysis/patholoscope/).

Besides, we calculated the microvessel density (MVD) and lymphatic microvessel density (LMVD) values of the specimens of the intratumoral area. MVD was defined as the number of blood vessels per unit area of tumor tissue (immunohistopathological images of the CD31 were used), while LMVD was defined as the number of lymphatic vessels per unit area (immunohistopathological images of the D2–40 were used).

### Statistical analyses

Appropriate statistical analyses were performed on the extracted data. Statistical analyses were performed using the non-parametric Mann-Whitney U test, Spearman correlation coefficient, chi-square test, and a multivariate logistic regression analysis as appropriate for the data set. Differences were considered significant at *P* < 0.05. All statistical analyses were performed using IBM SPSS Statistics version 22 (IBM Corp., Armonk, NY, USA).

## Results

**Fig. 1:**
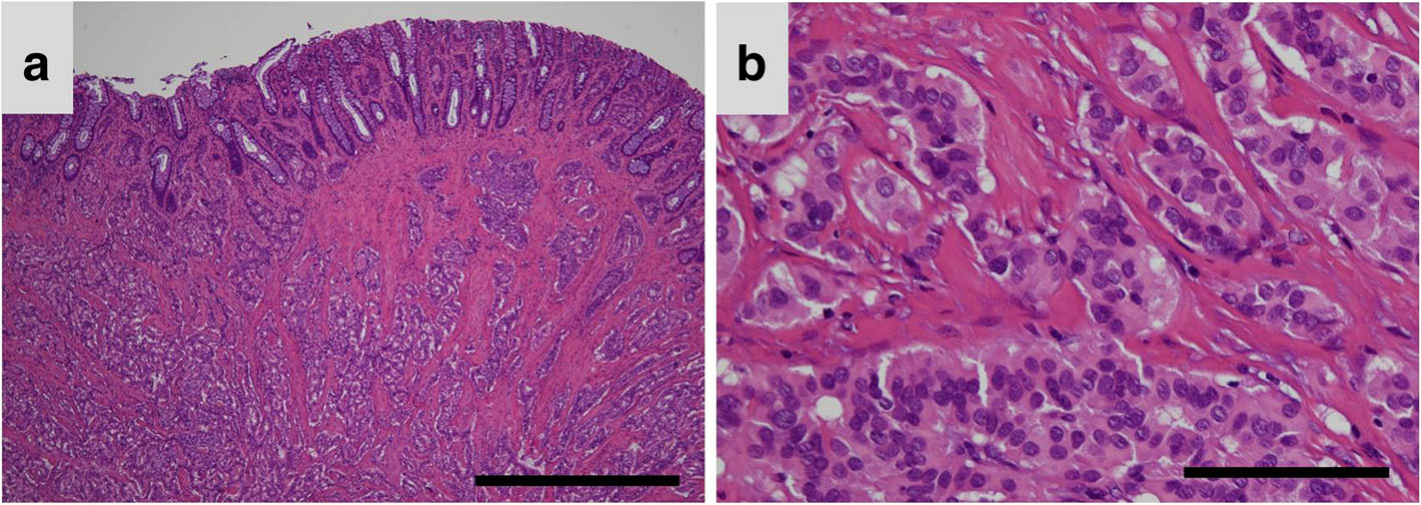
Representative images of histopathological findings in hindgut neuroendocrine tumors. **a** A photomicrograph showing a low-power field image of a hindgut neuroendocrine tumor (NET). The tumor cells are arranged in a trabecular pattern and show solid nests (Hematoxylin and eosin (HE) staining; original magnification, ×40; scale bar represents 1000 μm). **b** A photomicrograph showing a high-power field image of a hindgut NET. The tumor cells are uniform, arranged in rounded, solid nests, and have round-to-oval nuclei. Mild nuclear atypia can be seen (HE staining; original magnification, ×400; scale bar represents 100 μm)

Fifty-six cases with available FFPE specimens were analyzed (Fig. 1). Clinicopathological data are summarized in Table 1. Fourty four patients underwent an endoscopic procedure for removal; the remaining 12 patients underwent a surgical procure. The mean age (± standard deviation: SD) was 59.5 ± 12.7 years (range, 27–84 years), with a male-to-female ratio of 5:3 (35:21). The follow-up period ranged from 11 months to 290 months. While relatively a large number of patients remain alive, 13 of 56 patients died from various diseases. Especially, one patient who presented with lymph node and liver metastasis died 36 months after surgery. The remaining 12 patients died from other diseases causes (four cases involving gastric cancer, individual cases involving cerebral hemorrhage, extrahepatic cholangiocarcinoma, malignant lymphoma, rectal adenocarcinoma, and small cell lung cancer and causes of death were unknown for three cases).

**Table 1:** Clinicopathological characteristics of participants with hindgut NET

Characteristics	
Age (years)	
Mean ± SD	59.5 ± 12.7
Range	27–84
Sex (*n*, %)	
Male	35 (62.5%)
Female	21 (37.5%)
The maximum tumor diameter (mm)	
Mean ± SD	7.7 ± 7.9
Range	2.2–50.0
Ki 67 labeling index (%)	
Mean ± SD	1.3 ± 1.1
Range	0.0–4.2
Venous invasion (*n*, %)	
Negative	39 (69.6%)
Positive	17 (30.4%)
Lymphatic invasion (*n*, %)	
Negative	39 (69.6%)
Positive	17 (30.4%)
MVD (mm^2^)	
Mean ± SD	32.0 ± 31.2
Range	1.4–136.9
LMVD (mm^2^)	
Mean ± SD	9.4 ± 10.9
Range	0.35–55.0

*NET* neuroendocrine tumor, *MVD* Microvessel density, *LMVD* Lymphatic microvessel density, *SD* Standard deviation

**Fig. 2:**
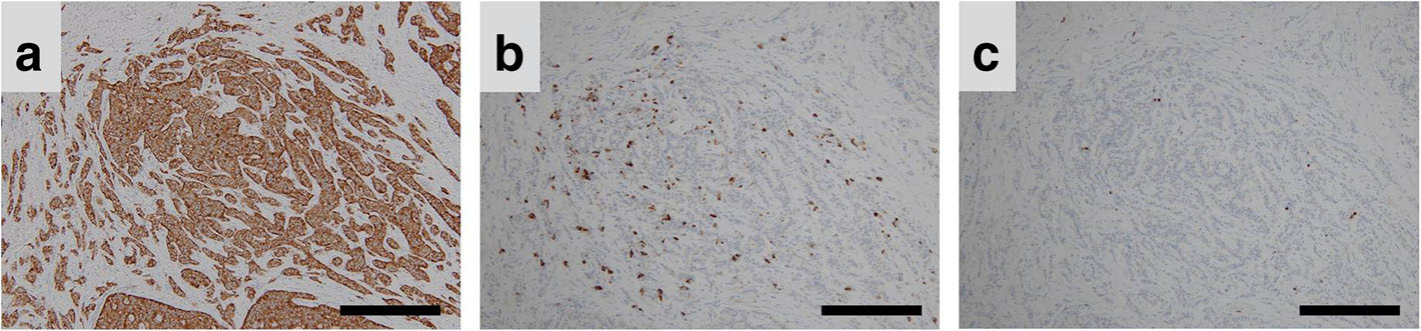
Immunohistochemical reactivity for synaptophysin, chromogranin A, and Ki-67 in hindgut neuroendocrine tumors. Representative photomicrographs of immunohistochemical staining. **a** Tumor cells showed strong positive reactivity for synaptophysin (original magnification, ×100; scale bar represents 300 μm). **b** Tumor cells showed sporadic positive reactivity for chromogranin A (original magnification, ×100; scale bar represents 300 μm). **c** A few tumor cells showed positive reactivity for Ki-67 (original magnification, ×100; scale bar represents 300 μm)

Pathological investigations revealed that 55 of 56 hindgut NETs were located in the rectum; the remaining NETs developed in the sigmoid colon. The mean maximum tumor diameter was 7.7 ± 7.9 mm (range, 2.2–50 mm). In 54 of 56 cases, the tumor invaded into the submucosal layer, and into the muscularis propria in the remaining two cases. Level 1 lymph node metastasis was observed in eight patients. Positive immunoreactivity for synaptophysin and/or chromogranin A was confirmed in all 56 cases (Fig. 2). The mean Ki-67 labeling index was 1.3 ± 1.1% (range, 0–4.2%, Fig. 2). Based on the Ki-67 labeling index, 41 and 15 cases were classified as NETs G1 and G2, respectively. Both venous and lymphatic invasion was identified in 17 cases each (30.4%). Mean MVD was 32 ± 31.2/mm^2^ (range, 1.4–136.9/mm^2^), and mean LMVD was 9.4 ± 10.9/mm^2^ (range, 0.35–55/mm^2^).

### Risk factors for metastasis

**Fig. 3:**
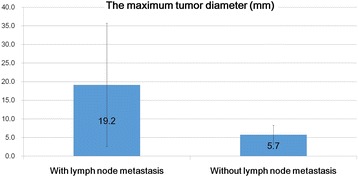
Differences in the maximum tumor between tumors with and without lymph node metastasis. The maximum tumor diameter in patients with lymph node metastasis was significantly larger compared with that in those without lymph node invasion. The maximum tumor diameter was a significant risk factor for lymph node invasion in hindgut neuroendocrine tumors

In the present study, because distant metastasis was found in one patient alone, who eventually died because of the NET, it was not possible to determine the prognostic impact of distant metastasis as a risk factor. Therefore, lymph node metastasis was evaluated as indirect evidence for risk factors associated with metastasis. In the univariate analyses, the maximum tumor diameter (Mann-Whitney U test, *P* < 0.001, Fig. 3), venous invasion (Mann-Whitney U test, *P* = 0.033), and MVD (Mann-Whitney U test, *P* < 0.001) were significant risk factors for lymph node metastasis in hindgut NETs. Multivariate logistic regression analysis (Table 2) revealed that the maximum tumor diameter was an independent predictor of lymph node metastasis (odds ratio, 1.5; 95% confidence interval (CI), 1.04–2.15; *P* = 0.03). By contrast, venous invasion (odds ratio, 0.27; 95% CI, 0.02–4.40; *P* = 0.36) and MVD (odds ratio, 1.04; 95% CI, 1.00–1.08; *P* = 0.08) were not independent risk factors for lymph node metastasis.

**Table 2:** Multivariate logistic regression analysis of lymph node metastasis

Variables	Odds ratio	95% CI		*P*-values
		Lower boundary	Upper boundary	
Tumor size	1.50	1.04	2.15	0.03
Venous Invasion	0.27	0.02	4.40	0.36
MVD	1.04	1.00	1.08	0.08

*CI* confidence interval, *MVD* micro vessel density

### Practical implications of MVD and LMVD

**Fig. 4:**
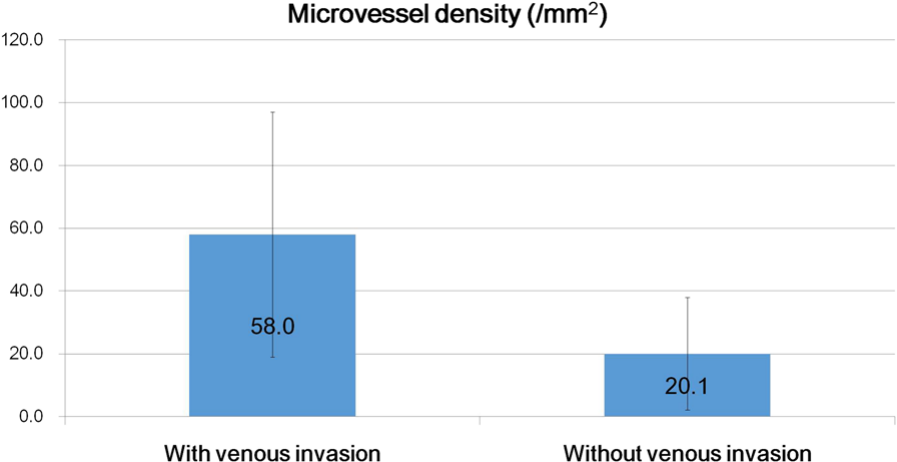
Differences in microvessel density between tumors with and without venous invasion. The microvessel density (MVD) in tumors with venous invasion was significantly higher compared with that in tumors without venous invasion. MVD was a significant risk factor for venous invasion in hindgut neuroendocrine tumors (Mann–Whitney U test, *P* < 0.001; values are expressed as the mean ± standard deviation)

**Fig. 5:**
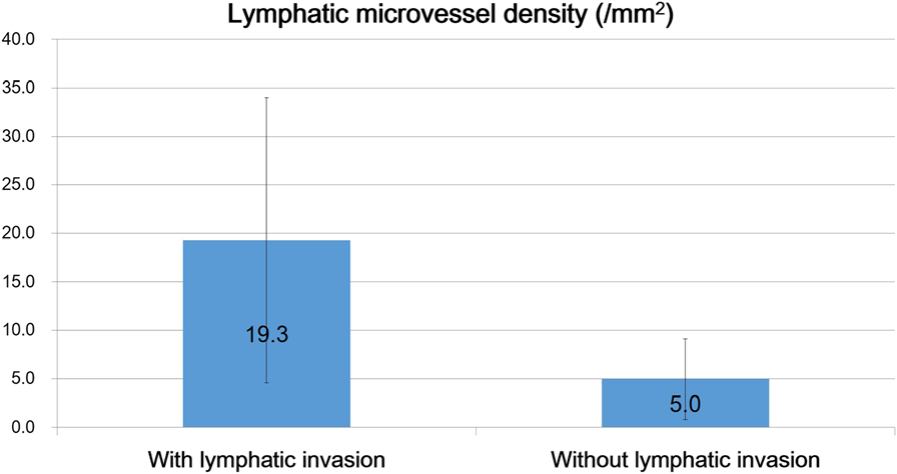
Differences in lymphatic microvessel density between tumors with and without lymphatic invasion. The lymphatic microvessel density in tumors with lymphatic invasion was significantly higher compared with that in tumors without lymphatic invasion. LMVD was a significant risk factor for lymphatic invasion in hindgut neuroendocrine tumors (Mann–Whitney U test, *P* < 0.001; values are expressed as the mean ± standard deviation)

**Fig. 6:**
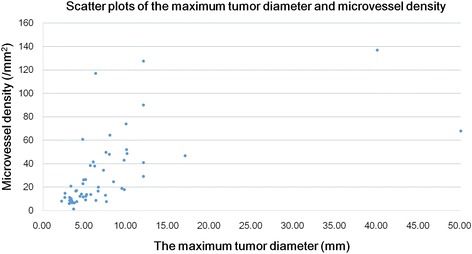
Scatter plots of the hindgut neuroendocrine tumor between the maximum tumor diameter and microvessel density. A significant positive correlation was found between microvessel density and the maximum tumor diameter (*r* = 0.735, *P* < 0.001, Spearman correlation coefficient)

MVD values were higher in tumors with venous invasion (mean, 58 ± 38.9/mm^2^) compared to those without venous invasion (mean, 20.7 ± 17.9/mm^2^; Mann-Whitney U test, *P* < 0.001; Fig. 4). LMVD values were higher in tumors with lymphatic invasion (19.3 ± 14.7/mm^2^) compared to those without lymphatic invasion (mean 5.0 ± 4.1/mm^2^; Mann-Whitney U test, P < 0.001; Fig. 5). Therefore, in hindgut NETs, MVD and LMVD could be considered as significant risk factors for venous and lymphatic invasion, respectively. Moreover, there was a positive correlation between the maximum tumor diameter and MVD (*r* = 0.735; Spearman’s correlation coefficient, *P* < 0.001; Fig. 6).

### Practical implications of the Ki-67 labeling index

In the present study, there were no significant correlations between the Ki-67 labeling index and any of the parameters evaluated (i.e., age, sex, the maximum tumor diameter, venous invasion, lymphatic invasion, MVD, LMVD, and lymph node metastasis).

## Discussion

Recently, an increased incidence of GEP-NETs has been reported globally, with the rectum, considered as the “intestine” of the hindgut, being the most common site of occurrence [1, 12]. Therefore, elucidating the mechanisms of hindgut NET progression and metastasis is important, and this study was specifically conducted to evaluate the risk factors associated with tumor progression and metastasis in hindgut NET.

In the univariate analyses, the maximum tumor diameter, venous invasion, and MVD were determined as significant risk factors for lymph node metastasis in hindgut NET. The maximum tumor diameter and the presence of lymphovascular invasion are generally known as important predictive factors for any tumor [3, 13–21]. However, results of our multivariate logistic regression analysis of lymph node metastasis revealed that the maximum tumor diameter alone was an independent predictor of lymph node metastasis, whereas lymphovascular invasion and MVD was not the predictor of lymph node metastasis. This finding indicated that the most important factor in the clinical management of patients with hindgut NET is the maximum tumor diameter. Actually, approximately 30% of patients had the lymphovascular invasion, but there were no significant correlations between lymphovascular invasion and lymph node metastasis. In general, additional surgery is recommended if the lymphovascular invasion was detected in endoscopically resected specimens of hindgut NET [22–24]. However, our data indicated that the lymphovascular invasion in endoscopically resected specimens of hindgut NET might not be the absolute indication for additional surgery. In fact, other investigators also advocated that further studies need to determine whether additional surgery is necessary or not for patients who are detected lymphovascular invasion in endoscopically resected specimens [25–27]. Although our study has not yet denied the pathological significance of lymphovascular invasion, it raises the question of whether the presence of lymphovascular invasion, in endoscopically obtained hindgut NET tissues, is an absolute indication for additional surgery or not.

Meanwhile, what is intriguing for us is that no significant correlations were identified between the Ki-67 labeling index and any parameter (age, sex, the maximum tumor diameter, venous invasion, lymphatic invasion, MVD, LMVD, and lymph node metastasis). In general, Ki-67 labeling index is regarded as a prognostic factor for many neoplasms [7, 28–33]. However, we wish to emphasize that Ki-67 labeling index is not an absolute prognostic factor in hindgut NET cases with the relatively low level of proliferative activities.

Regarding the morphometric analyses of MVD and LMVD, further discussion is warranted because previous studies have reported that NETs usually have a high MVD [34]. A high MVD would imply that NETs possess substantial angiogenic activity. Besides, because there was a positive correlation between MVD and the maximum tumor diameter in the present study, one could conclude that an angiogenic mechanism plays a major role in the progression of hindgut NET. Furthermore, since MVD was a significant risk factor for venous invasion, tumor progression and high MVD might be associated with hematogenous metastasis. Therefore, molecular, biological, and genetic analyses [35–38] of factors such as the angiogenesis-related genes could provide the key to elucidating the mechanisms of hindgut NET progression and/or metastasis.

By contrast, although LMVD was a significant risk factor of lymphatic invasion, no significant correlation was identified between LMVD and lymph node metastasis in the present study. Similarly, a previous study in patients with breast cancer failed to find any significant correlation between LMVD and lymph node metastasis [39]. Under certain circumstances, tumor progression might destroy the lymphatic vessels resulting in a subsequent decrease in LMVD. Thus, the pathologist should be aware of false-negative results in the assessment of lymphatic invasion in hindgut NET, despite there are many questions regarding the pathological significance of lymphovascular invasion. However, the limitations of our study need to be considered in the interpretation of our results. Foremost, this is a retrospective case series and relatively small sample size, therefore, are subject to the inherent biases.

## Conclusion

Since a positive correlation was identified between MVD and the maximum tumor diameter, angiogenic pathways may play a major role in the progression of hindgut NET. Therefore, molecular, biological, and genetic analyses of factors such as the angiogenesis-related factors could provide the key to elucidate the mechanisms of hindgut NET progression and/or metastasis.

Otherwise, a multivariate logistic regression analysis of lymph node metastasis revealed that the maximum tumor diameter alone was an independent predictor of lymph node metastasis in hindgut NET.

Moreover, although our study has not yet denied the pathological significance of lymphovascular invasion, it raises the question of whether the presence of lymphovascular invasion, in endoscopically obtained hindgut NET tissues, is an absolute indication for additional surgery or not.

## Abbreviations

FFPE: Formalin-fixed paraffin-embedded
GEP-NETs: Gastroenteropancreatic neuroendocrine tumors
LMVD: Lymphatic microvessel density
MVD: Microvessel density
NETs: Neuroendocrine tumors
SD: Standard deviation
WHO: World Health Organization
